# Sexual violence in the protracted conflict of DRC programming for rape survivors in South Kivu

**DOI:** 10.1186/1752-1505-3-3

**Published:** 2009-03-15

**Authors:** Birthe Steiner, Marie T Benner, Egbert Sondorp, K Peter Schmitz, Ursula Mesmer, Sandrine Rosenberger

**Affiliations:** 1 Independent Researcher, Krefeld, Germany; 2 Malteser International, Cologne, Germany; 3 London School of Hygiene and Tropical Medicine, London, UK; 4 Malteser International, Bukavu, DR Congo; 5 Malteser International, Kinshasa, DR Congo

## Abstract

**Background:**

Despite international acknowledgement of the linkages between sexual violence and conflict, reliable data on its prevalence, the circumstances, characteristics of perpetrators, and physical or mental health impacts is rare. Among the conflicts that have been associated with widespread sexual violence has been the one in the Democratic Republic of the Congo (DRC).

**Methods:**

From 2003 till to date Malteser International has run a medico-social support programme for rape survivors in South Kivu province, DRC. In the context of this programme, a host of data was collected. We present these data and discuss the findings within the frame of available literature.

**Results:**

Malteser International registered 20,517 female rape survivors in the three year period 2005–2007. Women of all ages have been targeted by sexual violence and only few of those – and many of them only after several years – sought medical care and psychological help. Sexual violence in the DRC frequently led to social, especially familial, exclusion. Members of military and paramilitary groups were identified as the main perpetrators of sexual violence.

**Conclusion:**

We have documented that in the DRC conflict sexual violence has been – and continues to be – highly prevalent in a wide area in the East of the country. Humanitarian programming in this field is challenging due to the multiple needs of rape survivors. The easily accessible, integrated medical and psycho-social care that the programme offered apparently responded to the needs of many rape survivors in this area.

## Introduction

Today's armed conflicts mostly occur within state borders and typically drag on for years, even decades. Multicausal in nature, these crises are typically "highly politicised" and "frequently associated with non-conventional warfare" [1,2]. National accountability mechanisms are characteristically absent or severely weakened [3], which consequently gives rise to a climate of impunity for perpetrating all sorts of crimes. These conflicts tend to affect the civilian sphere, regardless of growing international emphasis on the protection of civilians in conflict. Civilians are affected accidentally as they are not well distinguishable from combatants, or intentionally. They may be intentionally targeted because "the goal of warfare is not simply the occupation and control of territory [anymore] [...] – it is about destroying the identity and dignity of the opposition" [4]. One of the strategies to achieve this goal is by targeting women's sexuality and reproductive capacity. Sexual violence, therefore, not only causes individual physical and psychological ill health and social exclusion, but uproots families and communities and contributes to the moral and physical destruction of society [5]. In the absence of governmental programmes to mitigate the impacts of sexual violence humanitarian organizations play a role in caring for rape survivors, as is the case in the Democratic Republic of the Congo (DRC). International humanitarian organisations support the Congolese government's efforts to address the issue of sexual violence, among them Malteser International, a non-governmental organization. Since 2003, Malteser works with rape survivors in the Eastern province South Kivu by offering medical and psycho-social care. Over the years a host of programme data were collected, forming a unique dataset in the light of the overall scarcity of data related to sexual violence and rape survivors. Here we present an analysis of these data against a background of a literature review on sexual violence in the DRC crisis and discuss implications for humanitarian programming.

## Background

More or less ongoing since 1996, the DRC is experiencing a prolonged conflict. It has been characterized by extreme violence, mass population displacements, widespread rape, and a collapse of public health services [6,7]. The total death toll (1998–2007) was estimated at 5.4 million [8]. The conflict keeps flaring up despite several attempted peace accords and the deployment of a UN peacekeeping force, MONUC (*Mission des Nations Unies au Congo*), the largest UN peacekeeping operation with a strength of 18,000 uniformed personnel.

Sexual violence has not been sufficiently addressed by the DRC government, despite recent efforts such as the establishment of the *Ministry of Social Affairs and the Family *and initiation of a concerted initiative on sexual violence composed of NGOs, the United Nations and the Congolese Government. Sexual violence has further been included in the mandate of DRC's Truth and Reconciliation Commission. In July 2006 the Congolese Parliament passed the *Law on the Suppression of Sexual Violence *which anticipates strengthened penalties for perpetrators and more effective criminal procedures. Also, the DRC is party to several human rights treaties addressing women's rights, such as the Convention for the Elimination of All Forms of Discrimination against Women [9] and the Rome Statute of the International Criminal Court [10] which recognize sexual violence as both a crime against humanity and a war crime. However, DRC's juridical institutions remain weak and impunity for perpetrators largely prevails.

Humanitarian programming in the field of sexual violence is difficult. Not only is meeting the multiple needs of rape survivors a complex undertaking, the perpetual lack of data hampers programme evaluation and further research. There is great scarcity of data on the prevalence, circumstances, characteristics of perpetrators, and physical or mental health impacts. Several reasons contribute to this scarcity. Humanitarian programmes tend to allocate all available, usually scarce resources to directly address survivors' needs and pay less attention to data collection and research (see [11]). Furthermore, conflict situations impede structured research due to prevailing chaos and security threats for staff. Existing data are usually derived from project proposals and reports to donor agencies. Data thus collected may not be coherent and may not be easily compiled. Insufficient cooperation between humanitarian organisations may contribute to disintegration of data [12]. Data collection is further impeded by poor reporting of events. The majority of rape survivors resists speaking out for fear of social stigmatisation or denial ([13-16]). And if survivors do report sexual violence, it is often months or years after the incident, making a timely representation of sexual violence impossible. The UNHCR estimates that less than 10% of sexual violence cases in non-refugee situations are reported [17].

Care for rape survivors is complex, since it, ideally, comprises health care, psycho-social care, safety and legal aid so as to enable the survivors to institute proceedings against the perpetrators ([18,19]).

Timely access to health care is essential as sexual violence constitutes serious health threats for survivors. Sexually transmitted infections (STI's) are recognized consequences of rape [20], which need to be treated at an early stage [21]. The effectiveness of post-exposure prophylaxis (PEP) regarding possible HIV-transmission also depends on early initiation of therapy. As to inhibit unintended pregnancy, emergency contraceptive pills have proven to be effective in 56 – 94% of cases when taken within 120 hours of unprotected intercourse according to the World Health Organization [22]. Tetanus and hepatitis B vaccination should also be administered within 14 days of the incident unless the survivor was fully vaccinated [23].

Psychological support is regarded as another important aspect of rape care, as psychological effects of sexual violence are manifold and potentially last for a lifetime. About half of female rape survivors develop clinical symptoms of Post-Traumatic Stress Disorder (PTSD) at some point in their lives [24]. Other psychological manifestations include anorexia/bulimia nervosa, depression, and anxiety ([25,26]). During conflict, the psychological distress of rape survivors can be greatly aggravated by the breakdown of usual support systems and by the absence of a safe and supportive environment for healing [27]. Psychological care aims at stabilizing the survivor emotionally and mediating social relationships. Considering the common cultural background, local women seem best for providing psychological support and mediation between family members. Awareness-raising among the public is another pillar of programme design and focuses on eliminating prejudices and lowering discrimination against rape survivors. Simultaneously, strategies are promoted that aim at educating women on how to avoid risky situations, for example fulfilling chores in a group of females rather than alone, about available rape care and the importance of seeking medical care as soon as possible.

## Methodology

For this article, we analysed a number of data sets collected within Malteser International's medico-social programme for rape survivors in South Kivu, Eastern DRC.

The organisation provides medical treatment at specialized health centres [so called VAS-centres (*victimes d'aggression sexuelle*)] and psycho-social care through local community-based organizations (CBOs). Preventional aspects, i.e. awareness-raising among the public and information about sexual violence and available support, are also integral part of the programme. All data we used have been extracted from project proposals and reports written as part of project documentation to the donor, the *European Commission's Department for Humanitarian Aid *(ECHO). Under the difficult operating circumstances, data were not always collected consistently. While we have data from the initiation of the Malteser programme in 2003 to the final report of December 2007, data for 2003 and 2004 are not accurate enough to be included in the analysis. For the years 2005–2007 we could retrieve data on the number of attended rape survivors, place of origin, treatment provided, referral rates and numbers of women rejected by their families and consequent successful reintegration. Furthermore, for the period October to December 2005 we found specific data on *age distribution *and *time between rape and medical attendance*.

Data on rejected rape survivors derive from collaborating local community-based organisations (CBOs), which carry out family mediation when women have been rejected by their families as a consequence of sexual violence.

## Results

**Figure 1:**
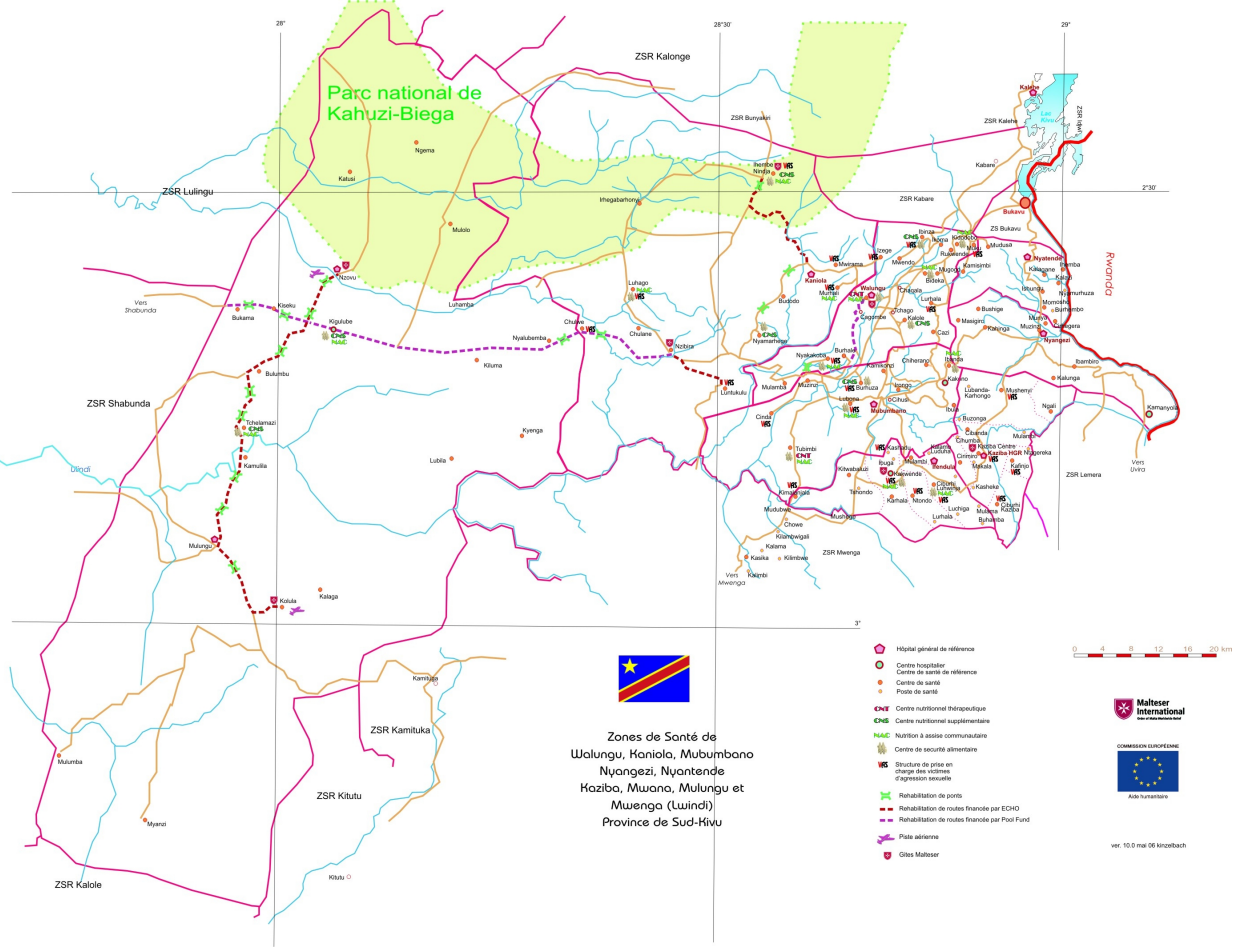
Location of VAS-centres in South Kivu, DRC.

The Malteser programme for rape survivors includes three approaches: Medical care, psycho-social support, and awareness-raising (Table 1). Survivors receive medical care free of charge in one of 23 Malteser-supported health centres that are specialized in the treatment of sexually abused women (VAS-centres) [for a detailed map on location of VAS-centres in South Kivu, DRC, see figure 1]. They have specially trained nurses on site who perform rape-related diagnostics (i.e. HIV- and pregnancy tests) and provide medical treatment for conditions related to sexual violence such as wounds and sexually transmitted infections (STI). Since medical treatment in the VAS-centres is basic, women with more complicated health conditions (mostly drug-resistant STIs) are sent to one of the reference hospitals in the region for secondary care. Women requiring advanced surgery (for example for treatment of fistula-repair) are referred to the Panzi Hospital in Bukavu, a non-for-profit hospital specialised in repair of genitourinary fistula secondary to sexual violence (Table 2 and table 3).

**Table 1:** General information on Malteser International rape survivors' programme in South Kivu, DRC

• Total population of South Kivu, DRC 963,000 (2006 est.)
• Population with access to VAS-centres: approx. 202,600 (2006 est.)
• Approx. 100 health centres located in 9 health zones
• located in one of 5 health zones: (Walungu, Kaziba, Mumumbano, Kaniola, Mwana)
• 18 Community Based Organizations (CBO's) provide psycho-social care
• Programme started in 2003 when during a period of intense fighting in South Kivu over 1,000 women were registered with sexually transmitted infections resulting from rape
• About 20,517 cases of rape (registered January 2005–December 2007)

**Table 2:** Medical services offered to rape survivors through Malteser

• Medical care and psycho-social assistance
• Integrated into local health structures
• Medical treatment at specialized health centres (VAS-centres)
• Psycho-social counselling through 18 local CBOs
(community-based organizations); about 10 local staff each
• Curriculum: 2 day-teachings in psycho-social assistance/
basic medical knowledge, monthly supervisions
• Malteser pays running costs and staff salaries
• Salary per staff: 35 USD/month plus variable additional payment based on performance (97 – 145 USD/month)
• Awareness-raising about sexual violence through Provincial Health Inspection with special focus on combatants

**Table 3:** Characteristics of Malteser VAS- programme

• Presumptive treatment for STIs within 2 weeks after incident, otherwise symptom-oriented
• PEP (post-exposure prophylaxis) within 72 hours (in 4 VAS-centres)
• HIV counselling
• Anti-retroviral treatment in cooperation with MSF (only in Bukavu)
• Pregnancy tests, special programme for women with rape-related pregnancies
• referral system for advanced medical treatment (e.g.: operations)

The psycho-social support programme is carried out by 18 local CBOs while each has about 10 active local staff members to provide individual and group counselling and family mediation/home visits. Survivors are either referred from the VAS-centres to local CBOs for psycho-social support, or are enrolled on their own initiative. In cases survivors have been rejected by their husbands and families, CBO staff engages in dialogues with families and survivors, trying to eliminate prejudices about rape.

Awareness raising campaigns and health education as integral part of the programme are mainly implemented by the *Provincial Health Inspection*. Women receive information on how to find medical and psychological care and about the importance of seeking timely medical help when having been assaulted. Additionally, and as a preventive strategy, awareness-raising and education for combatants aim at reducing the incidence of rape. Education at the community level seeks to eliminate stigmatization of violated women and facilitate re-integration into society. Key messages are basic and include the facts that it is not the woman's fault to be raped, and that transmitted diseases can be diagnosed and (mostly) be cured. Information for the general public are mainly conveyed via radio and newspaper. The *Provincial Health Inspection *also holds regular meetings with civil authorities, other leaders of the communities and the military to campaign against sexual violence and fight impunity.

In table 4 we provide an overview of 2005–2007 data. For the year 2005 data were most complete. During the year 2006 and 2007 some data were missing or we could only find percentages, not the absolute numbers. If not indicated otherwise, the last column holds more explanation on this data.

**Table 4:** Programme findings

**Indicators**	**2005**	**2006**	**2007**	**Remarks**
Number of identified survivors	9,109	6338	8541	only rape (penetrative sexual assault)
STI treatment	5,987 (66%)			38% *partner treatment in 2006, 45% in 2007*
HIV test	133 (1,5%)	58%	57%	
Referral to secondary hospitals	242 (3%)			Advanced medical treatment, e.g. drug-resistant STI
Referral to hospital Panzi (Bukavu)	29 (0,3%)			Advanced surgical treatment, e.g. fistula repair
Time between rape and Malteser registration				Reasons: Fear, stigmatisation, shame; kidnappings
72 h	3,2%	151 (2,4%)	47 (0,6%)	
< 1 month	7,8%			
< 1 year	59,0%			
Psycho-social treatment	4099 (45%)	5440 (86%)	7536 (88%)	Home mediation visits, individual and group counselling
Rejection by family/social exclusion	506 (12,4%)	approx. 6%	208 (2,4%)	
Reintegration into families	310 (61%)	approx. 65%	37%	
Costs	215,000€ (23 €/case)			Additionally: Prevention & Advocacy (ca. 1 €/capita)

### Number of identified rape survivors

Between January 2005 and December 2007 a total of 20,517 rape survivors have been registered with the Malteser programme in South Kivu province. Table 5 shows a breakdown by the five health zones.

**Table 5:** Number of Malteser-registered rape survivors (2005–2007) in the 5 health zones by year, South Kivu, DRC

**Zones**	Walungu (164,509 Pop.)	Kaniola (125,496 Pop.)	Mubumbano (118,247 Pop.)	Kaziba (91,026 Pop.)	Mwana (114,895 Pop.)	**TOTAL** **(614,173 Pop.)**
Identified rape survivors (2005)	996	3,252	978	639	3,244	**9,109**
Identified rape survivors (2006)	1,180	2,208	876	708	1,366	**6,.338**
Identified rape survivors (2007)	1,134	1,863	755	267	1,051	**5,070**

### Time between rape and medical attendance

**Figure 2:**
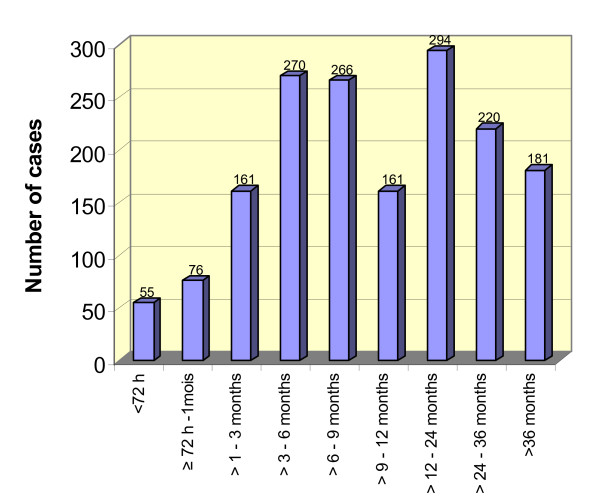
Time between rape and medical attendance, October-December 2005.

For October-December 2005 we found details on the time between rape and medical attendance, showing that few women sought medical care within the first month, even less within the "critical" 72 hours after the incident (see figure 2). More than one third of patients had been sexually violated one year or longer ago. For the years 2006 and 2007 we only have the percentage of women that sought care within 72 hours (see table 4).

### Age distribution among rape survivors

**Figure 3:**
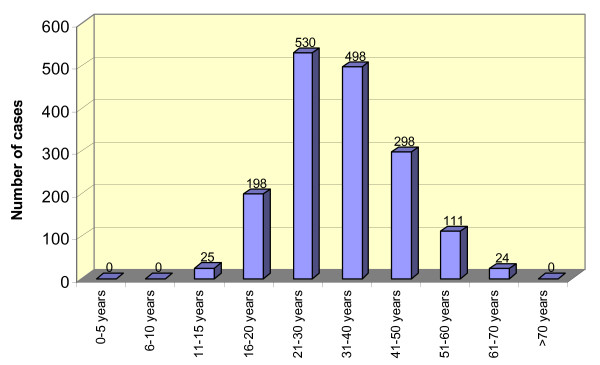
Age distribution among rape survivors.

Figure 3 demonstrates that women of all ages are targeted by sexual violence.

### Rejection of rape survivors by their families

Rejection of sexually assaulted women takes place rather frequently throughout the five health zones. The percentage of women being expelled from their homes after experiencing sexual violence fell from 12.5% in 2005 (table 5) to 6% in both 2006 and 2007. With 4 out of 10 rejected rape survivors, re-integration into the family failed despite family mediation (see table 6).

**Table 6:** Rape survivors rejected by families and successfully reintegrated by area (2005)

**Zone**	**# Identified rape survivors**	**# Rejected survivors** **(% of identified survivors)**	**# Reintegrated survivors** **(% of rejected survivors)**
Walungu	1118	52 (5%)	30 (58%)
Kaziba	395	93 (23%)	50 (54%)
Mumumbano	786	74 (9%)	46 (62%)
Kaniola	517	141 (27%)	75 (53%)
Mwana	1258	146 (12%)	109 (75%)
**TOTAL**	**4074**	**506 (12%)**	**310 (61%)**

## Discussion

Malteser data have been extracted from project proposals written as part of project documentation and have not been specifically generated as a basis for scientific examination. And even for the better documented period 2005–2007 not all data were consistently available. This also applies to the majority of available articles on sexual violence in the DRC where authors refer to data from humanitarian programmes or hospital registers ([28-30]). Individual testimonies are frequently cited in order to emphasise the pervasiveness of sexual violence in this conflict ([31-33]). Therefore, interpretation of the presented data should be done with caution.

We believe that despite this rather large cohort of women that was seeking care at the Malteser clinics, the majority of rape survivors have remained unidentified. For fear of social stigmatization by the family, socio-economic exclusion at the community level and/or repercussions by perpetrators many women resist to reveal the incident ([34-37]). Malteser data exclusively comprise women who were willing and able to disclose the incident to this organization. Considering that, despite these obstacles, about 20,500 rape survivors were identified by Malteser within 36 months elucidates the pervasiveness of sexual violence in the DRC conflict. Consequently it is highly likely that numbers in reality are much higher. This becomes particularly apparent in a recent Malteser survey that found that 73% of South Kivu residents knew someone who was a rape survivor [38].

Our data demonstrate that women of all ages are targeted by sexual violence (see figure 3). This is congruent to the findings of *Réseau des Femmes pour un Développement Associatif *et al. (RFDA) [39] and Pratt and Werchick [40], with rape survivors ranging in age from 12 to 70 years, and 4 months to 84 years, respectively. This broad age span of survivors results in complex requirements for programmes which need to balance the needs of women throughout the life cycle.

We found that sexual violence in DRC frequently leads to social exclusion. Malteser data reveal that, in 2005, 12% of women were expelled from their home, mainly by the husband. Compared to other reports, this is a comparatively small percentage. For example, RFDA found that the percentage of DRC survivors abandoned by their husbands amounted to 26 percent [41]. The fact that in subsequent years of Malteser observation (namely 2006 and 2007), fewer women were expelled from their homes after they had become survivors of rape may signify success of the awareness-raising campaigns, which aim at lowering public stigmatization and discrimination against rape survivors. Another possible reason is that so many women throughout the society have been sexually assaulted that discrimination against them lessens. We also present reintegration data of initially rejected rape survivors. However, this may not mean a full return to the pre-existing situation. A woman was counted as having been successfully reintegrated if she could return home and live amongst her family again. The degree to which these living conditions were comparable to the time *before *the incident needs to be explored more in depth in the future. For now it remains unclear whether survivors are still discriminated against after they return, and whether their living conditions and social relationships are comparable to before the incident.

We found a decline in registered rape survivors in 2006/2007 vis-à-vis 2005. This is associated with the fewer numbers of "old" cases of rape that have been treated in the VAS-centres from 2006 on. Because specific services for rape survivors had been unavailable before, after initiation of the Malteser programme many survivors attended services who had experienced sexual violence several years ago. In 2005 the rape of 24% of programme attendees dated back two years or longer (see figure 2). In 2006, however, rape survivors with old conditions had mostly been taken care of and the majority of programme attendees comprised women the rape of which dated back less than one year [42]. This phenomenon of a drop in numbers some time after a clinic gets established was also described by Pratt and Werchick (2004): "The hospital staff warned us that the current increases [of rape survivors being seen in a DRC clinic] almost certainly do not represent new cases, but patients who were attacked months or even years ago and are coming for assistance only now as security allows and as more victims hear of services that have become available" [43].

Numbers of women that attended Malteser services within 72 hours after experiencing sexual violence are low and have not been increasing over the years. While, in 2005, 3,2% of rape survivors received care within 72 hours, the percentage dropped to 0,6% in 2007. Women seem not able to access VAS-centres in time. Possible reasons include insecurity in the area, fear of stigmatization and lacking awareness about the importance of receiving timely medical treatment. Several incidents of kidnappings have also been reported that typically last several days to months and hinder women from receiving proper care until after they are released.

Another limitation of our data is the lack of accurate information about the perpetrators of sexual violence. As RFDA (2005) noted, "trying to identify with any precision the perpetrators of the rapes and sexual violence is an impossible task" [44]. However, Malteser reports between 2005 and 2007 indicate a strong correlation between ongoing violent conflict in an area and the number of women seeking rape care. In a recent field study by Malteser International, 94% of interviewed South Kivu inhabitants stated that the rape they witnessed had been perpetrated by "the military or a (para)military group" [45]. This is congruent to the findings of several humanitarian organizations which have indicated that combatants may be the main and frequent perpetrators of sexual violence in DRC ([46-51]).

## Conclusion

We have documented that in the DRC conflict, rape has been – and continues to be – highly prevalent in a wide area in the east of the country. In 36 months (January 2005 – December 2007) about *20,500 *female Congolese rape survivors have been identified by Malteser International in South Kivu province. Considering the many obstacles that prevent rape survivors from reporting the incident, the real scope of sexual violence is likely to be manifold higher.

Data from the Malteser programme for rape survivors imply that, from 2006 on, numbers of programme attendees have declined while simultaneously there has been a shift in favour of early case reporting. As needs of rape survivors in the area had not been addressed before, we believe that the decline in programme attendance is due to abating numbers of "old" cases of sexual violence over the course of many months rather than signifying lesser frequency of sexual violence.

As our data demonstrate that women of all ages are targeted by sexual violence, we believe that programmes for rape survivors need to address the needs of women throughout the life cycle.

Sexual violence creates a significant risk for social – especially familial – exclusion. As demonstrated by our findings (2005), one in eight women was outcast by her family after experiencing sexual violence; more than every third of these had not been allowed to return home. Psycho-social counselling comprises an integral part of programming which is ideally carried out by local women with similar cultural background.

In line with other studies, we suggest that the main perpetrators of sexual violence in South Kivu, DRC, are military and paramilitary groups. Awareness-raising campaigns and lobbying against sexual violence at the community level with special focus on militarized groups encompass essentials of humanitarian programming that need to be reinforced.

There is great scarcity of data on sexual violence which hampers programme evaluation and further research. Therefore, we suggest that more emphasis should be paid on proper data collection and analysis.

Humanitarian programming in the field of sexual violence is challenging due to the manifold needs of women who have experienced sexual violence. Access to rape survivors continues to be difficult to acquire. As a precondition, medical services and psycho-social care need to be well established and easily to access at primary care level. We believe that rape care should preferably be provided through local groups and local health centres, and that international organizations may support the local health and social structures through expertise and finance. Trust building and networking are preconditions in addressing this public health problem.

## Competing interests

The authors declare that they have no competing interests.

## Authors' contributions

BS conceptualized and designed the case study, carried out the literature review, evaluated the data, drafted the initial manuscript and is principal author. MTB supported the concept and design of the study and participated in the writing process. ES contributed to all sections and edited the manuscript. KPS together with UM and SR provided the data and edited the first draft. All authors revised and approved the final text.
